# Coronary computed tomography angiography study on the relationship between the Ramus Intermedius and Atherosclerosis in the bifurcation of the left main coronary artery

**DOI:** 10.1186/s12880-023-01009-2

**Published:** 2023-04-11

**Authors:** Dan-Qing Zhang, Yan-Feng Xu, Ya-Peng Dong, Shu-Jing Yu

**Affiliations:** 1 Hebei Medical University, 050000 Shijiazhuang, China; 2 Department of Diagnostic CT, Cangzhou Central Hospital, No.16 of Xinhua West Road, Canal District, 061000 Cangzhou, China

**Keywords:** Ramus intermedius, Bifurcation angle, Coronary computed tomography angiography, Atherosclerosis

## Abstract

**Objective:**

This study aimed to explore the relationship between the ramus intermedius (RI) and atherosclerosis in the bifurcation of the left coronary artery (LCA).

**Methods:**

Screening patients who underwent CCTA from January to September 2021, 100 patients with RI (RI group) and 100 patients without RI (no-RI group) were randomly enrolled, Evaluation of RI distribution characteristics and left main coronary artery(LM),Left anterior descending branch(LAD),left circumflex branch(LCX) proximal segment plaque distribution, measurement of LAD-LCX bifurcation angle(∠LAD-LCX),Comparison of the three distribution characteristics with the incidence of plaques in the left main trunk bifurcation area (LM, LAD, LCX) between groups and within the RI group.

**Results:**

The difference in the incidence of plaques in the proximal LCX and the LM between the RI group and the no-RI group were not statistically significant (P > 0.05). The incidence of plaques in the proximal LAD in the RI group was significantly higher than that in the non-RI group (77% versus 53%, P < 0.05). However, there was no statistically significant difference between the two groups after PSM. A univariate logistic regression analysis revealed that an RI was a risk factor for plaque formation in the proximal LAD (P < 0.001), and a multivariate logistic regression analysis revealed that an RI was not an independent risk factor for plaque formation in the proximal LAD (P > 0.05). When compared within the RI group, the difference in the incidence of plaques in the proximal segment of LAD, the proximal segment of LCX, and the LM among the different distribution groups of RI was not statistically significant, respectively (P > 0.05).

**Conclusion:**

RI is not an independent risk factor for atherosclerosis in the left coronary artery bifurcation zone, but it may indirectly increase the risk of atherosclerosis in the proximal segment of the LAD.

## Introduction

With the development of interventional radiology, the geometric parameters of left coronary artery (LCA) bifurcation zone have attracted a large amount of attention because it is highly related to the complex decision-making regarding treatment and the incidence of perioperative and postoperative adverse clinical events [1–3]. Additionally, the geometry of the blood vessels has been proven to be one of the causes of atherosclerosis. Studies suggest that atherosclerotic plaques tend to occur in high curvature or bifurcation vascular areas [4, 5], and the increased bifurcation angle of the LCA is considered to be an independent risk factor at the early stage for the formation of atherosclerosis in the included angle area [6, 7]. A ramus intermedius (RI) is a coronary artery branch that originates from the end of the left main coronary artery(LM) and lies between the left anterior descending branch (LAD) and the left circumflex branch (LCX). The existence of the RI changes the geometric structure of the bifurcation area of the left coronary artery, which may cause hemodynamic changes in the bifurcation area and subsequently affect the formation of atherosclerotic plaques in the proximal segment of the bifurcation area of the left coronary artery. The purpose of this study is to investigate the correlation between the presence and distribution of an RI and the formation of atherosclerotic plaques in the proximal segment of the left coronary bifurcation area through coronary computed tomography angiography (CCTA).

## Study contents and methods

### General data

A total of 4,866 patients who underwent CCTA at Cangzhou Central Hospital from January 1, 2021, to September 1, 2021, were selected, and from that group, 1,202 patients were reported to have an RI through the picture archiving and communication system report. After excluding patients according to the exclusion criteria, 100 patients were randomly selected by the computer random number method. The average age of the selected patients was 61.90 ± 10.43 years. In terms of gender, 57 were males and 43 were females. Another 500 patients were randomly selected from 3,664 non-RI cases during the same time period by the computer random number method. After excluding patients according to the exclusion criteria, 100 patients were randomly selected as the no-RI group (the sample size thus met the statistical requirements). The average age of the selected patients was 58.11 ± 11.53 years. In terms of gender, 44 were males and 56 were females. Exclusion criteria: (1) Metal implants (stents, pacemakers, etc.); (2) A history of coronary artery bypass surgery; (3) Organic heart disease (including heart valve implantation); (4) Coronary artery variation (such as anomalous origin of the coronary artery and coronary arteriovenous fistula); (5) Poor CCTA image quality (images missing, etc.); (6) Branches: ≥1 of the LAD, the LCX, and the RI were mural coronary arteries; (7) Non-dominant right coronary artery; (8) Non-single RI (Except for in the non-RI group); and (9) Incomplete clinical data.

### Examination methods and data acquisition

#### Examination methods

All patients were fasted for more than 4 h before examination, and they were given an intravenous indwelling needle. Electrocardiogram leads were connected to measure their heart rate, and breath holding training was performed. Those with a faster heart rate were given β-receptor blockers to stabilize the patient’s heart rate (ensuring that the patient’s heart rate was stabilized within 74 beats/min).

All patients were scanned with a 320-slice computed tomography (CT) machine (Toshiba Aquiline ONE), and the data were post-processed. The examinee was in a supine position, and their feet entered the machine first. Both arms were stretched straight and placed over their head. Three leads were applied to the bilateral subclavian sites and left rib lower margin, respectively, and the leads were connected with a machine to monitor real-time heart rate changes. Scanning was performed with the last breath of a single inhalation The scanning range was determined according to the anteroposterior and lateral view, from the lower part of the tracheal bifurcation to the diaphragm. The range covered the whole heart, which was 140–160 mm. A Mallinckrodt double-chamber high-pressure syringe was used to inject 60–65 ml of the non-ionic contrast agent iohexol at an injection rate of 4.5–5.0 ml/s. The volume and flow rate were adjusted according to the patient’s weight and vascular conditions. Then, 40 ml of normal saline was injected at the same rate. Monitoring started after 10 s of contrast agent injection. Observing the time–density curve, the left ventricle was chosen as the monitoring area. It was triggered manually, with a trigger threshold of 150 HU. Scanning parameters: The tube voltage was 120 kV, the scanning slice thickness and gap were both 0.5 mm, and the rotation speed was 0.35 s. All CT scans were performed in automatic regulation mode of the tube current during the cardiac cycle to reduce radiation dosages.

#### Image post-processing and measurement

**Fig. 1:**
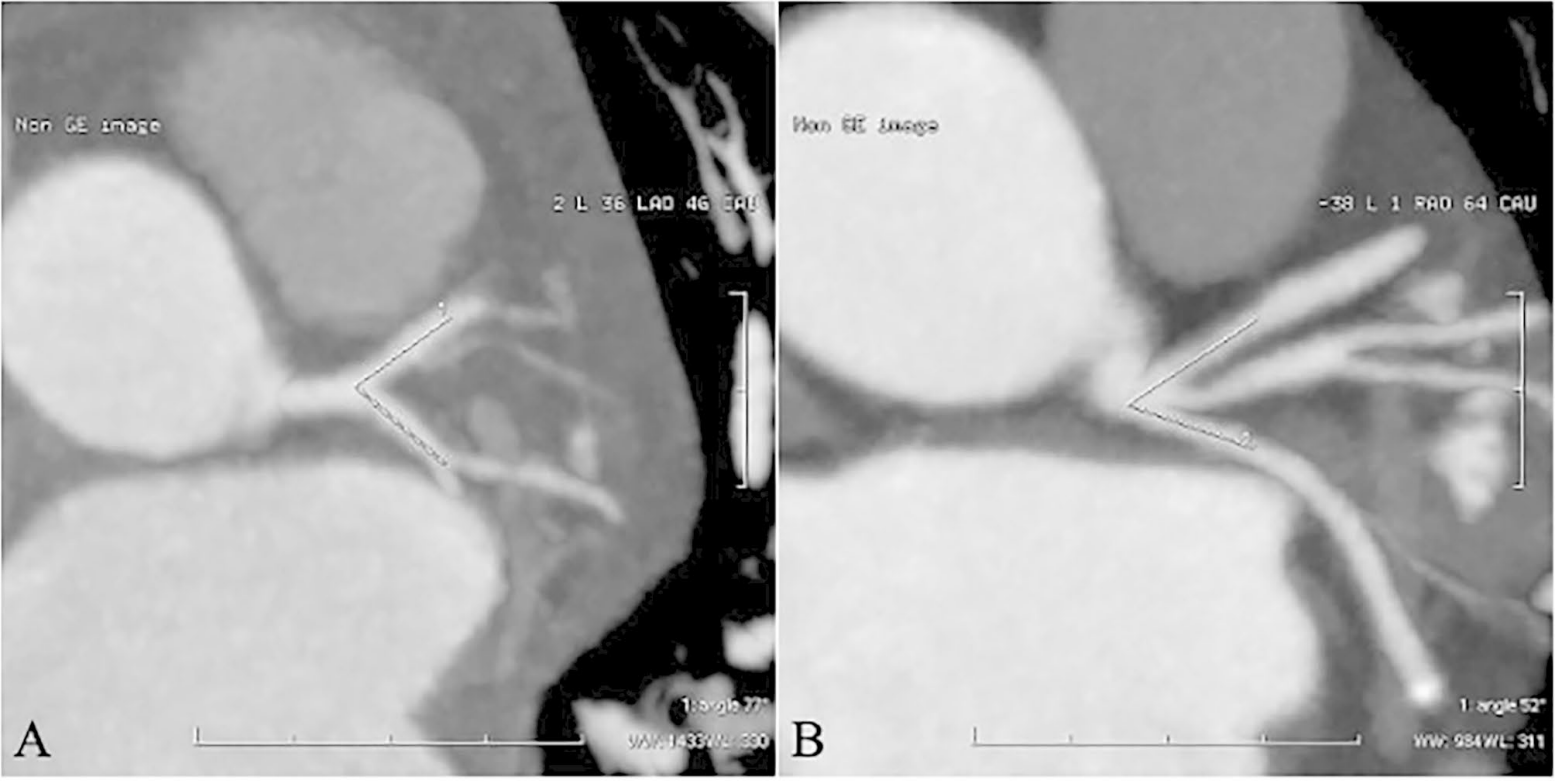
Measurement of ∠LAD-LCX angle (**A**: measurement without ramus intermedius **B**: measurement with ramus intermedius)

The best phase images were chosen and uploaded to a GE AW4.6 workstation. Next, two-dimensional (2D) and three-dimensional (3D) reconstruction images were generated through the Cardica CTA Analysis software. The proximal LM, LAD, and LCX plaques were observed from the 2D images. On the multi-plane maximum intensity projection (MIP) (MIP = 4 mm, window level (WL) = 40HU, window width (WW) = 400HU), the image was adjusted to display the maximum cross-sectional area of the oblique coronal plane of the proximal vessels of the LAD and LCX. An angle tool was used to measure the left trunk bifurcation angle (∠LAD–LCX). During the measurement, the intersection angle of the central axes of the LAD and LCX lumens on the saved image served as the intersection angle(∠LAD-LCX, Fig. 1); if the LAD or LCX was tortuous in the proximal segment, the lumen that was 10 mm away from the left trunk bifurcation was selected as the central axis [8].

#### RI distribution type definition

**Fig. 2:**
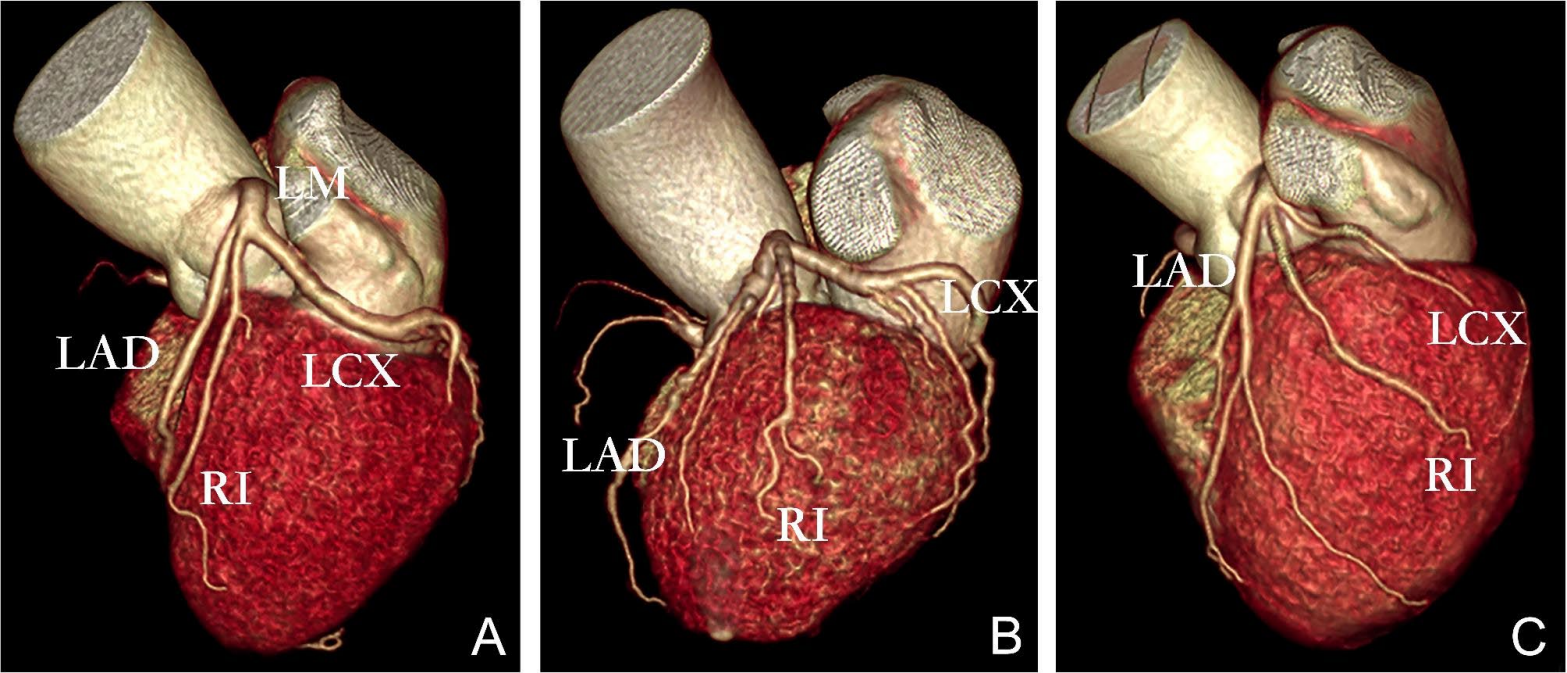
RI distribution (**A** shows distribution by interventricular groove, **B** shows centered distribution, C shows distribution by atrioventricular groove)

The RI distribution was determined by 3D volume rendering (Fig. 2). Taking the connecting line between the beginning and the apex of the heart as the boundary, the distribution of the RI was divided into three types. The first type was near the interventricular groove (near LAD group) (Fig. 2A). The second type was in the middle (middle group) (Fig. 2B). The third type was near the atrioventricular groove (near LCX group) (Fig. 2C).

#### Propensity score matching (PSM)

The study subjects were divided into RI group (n = 100) and no-RI group (n = 100), PSM was used for 1:1 fuzzy matching with a matching tolerance of 0.1, and a total of 52 pairs of study subjects were matched after equalizing the two groups in terms of age, diabetes, and angle differences.

### Statistics analysis

All data were statistically analyzed using statistical software SPSS 26.0. The normally distributed measurement data were expressed as the mean ± standard deviation (x ± SD), and the skewed distributed data were expressed as the median and interquartile range (M [IQR]). The data conforming to a normal distribution and homogeneity of variance were compared using a t-test and a one-way analysis of variance, otherwise they were evaluated using a nonparametric test. The count data were expressed as an n percentage (%) and compared between the groups using a chi-square test. A multivariate logistic regression analysis was performed to correct the confounding factors. A P value of < 0 0.05 was considered statistically significant. The inspection level was set at α = 0.05 (two-sided).

## Results

### The general data of the two groups and the comparison of the incidence of plaques in the bifurcation zone of the LCA

**Fig. 3:**
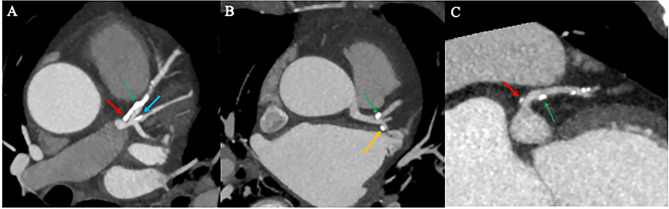
Plaque distribution (LM plaque (red arrow), LAD proximal segment plaque (green arrow), LCX proximal segment plaque (yellow arrow), RI proximal segment plaque (blue arrow))

There was no statistically significant difference between the pre-PSM RI group and the no-RI group in terms of gender, history of hypertension, history of hyperlipidemia, and history of smoking (P > 0.05, see Table 1). The differences between the two groups were statistically significant when comparing age, diabetes history, and ∠LAD-LCX angle (P < 0.05, see Table 1).The incidence of plaques in the proximal segment of the LAD (Fig. 3) was significantly higher in the RI group than in the no-RI group (P < 0.05, see Table 2);The RI group had a higher incidence of plaques in the proximal segment of LCX (Fig. 3) and LM (Fig. 3) than the no-RI group, but there was no statistical difference (P > 0.05, see Tables 2 and 3). There was no statistical difference in clinical baseline information between the two groups after PSM (P > 0.05, see Table 1), and no statistical difference in the incidence of LAD proximal segment, LCX proximal segment and LM plaque between groups (P > 0.05, see Table 2).Univariate logistic regression analysis showed that RI was a risk factor for plaque formation in the proximal segment of LAD (P < 0.001, see Table 4),After correction for confounding factors (age, history of diabetes, ∠LAD-LCX), RI was not an independent risk factor for plaque formation in the proximal segment of the LAD as shown in model 2 of the multifactorial logistic regression analysis (P > 0.05, see Table 4).

**Table 1:** Comparison of general clinical data before and after PSM in RI group and no-RI group

		Before PSM				After PSM	
	RI group (N = 100)	No-RI group (N = 100)	P		RI group (N = 52)	No-RI group (N = 52)	P
Age (years)	64.00(17)	57.00(16)	0.022^&^		61.94 ± 9.89	60.58 ± 11.12	0.510^*^
Gender (n%)							
male	57(57.0%)	44(44.0%)	0.066^#^		30(57.7%)	21(40.4)	0.078^#^
female	43(43.0%)	56(56.0%)			22(42.3%)	31(59.6%)	
hypertension (n%)							
yes	59(59.0%)	49(49.0%)	0.156^#^		31(59.6%)	26(50.0%)	0.325^#^
no	41(41.0%)	51(51.0%)			21(40.4%)	26(50.0%)	
diabetes (n%)							
yes	41(41.0%)	21(21.0%)	0.002^#^		15(28.8%)	13(25.0%)	0.658^#^
no	59(59.0%)	79(79.0%)			37(71.2%)	39(75.0%)	
hyperlipidemia (n%)							
yes	17(17.0%)	9(9.0%)	0.093^#^		7(13.5%)	6(11.5%)	0.767^#^
no	83(83.0%)	91(91.0%)			45(86.5%)	46(88.5%)	
smoke(n%)							
yes	17(17.0%)	13(13.0%)	0.428^#^		7(13.5%)	7(13.5%)	1.000^#^
no	83(83.0%)	87(87.0%)			45(86.5%)	45(86.5%)	
∠LAD-LCX(°)	90.50(33)	58.50(31)	< 0.001^&^		74.81 ± 20.64	75.92 ± 20.04	0.780^*^

*, Student’s t test. #, Chi-square test. &, Nonparametric test

**Table 2:** Comparison of plaque incidence in left coronary bifurcation before and after PSM in RI group and no-RI group

		Before PSM				After PSM	
	RI group (N = 100)	No-RI group (N = 100)	P		RI group (N = 52)	No-RI group (N = 52)	P
LM (n%)							
yes	34(34.0%)	25(25.0%)	0.163^#^		15(28.8%)	18(34.6%)	0.527^#^
no	66(66.0%)	75(75.0%)			37(71.2%)	34(65.4%)	
the proximal segment of the LAD (n%)							
yes	77(77.0%)	53(53.0%)	< 0.001^#^		35(67.3%)	27(51.9%)	0.110^#^
no	23(23.0%)	47(47.0%)			17(32.7%)	25(48.1%)	
the proximal segment of the LCX (n%)							
yes	35(35.0%)	24(24.0%)	0.088^#^		14(26.9%)	16(30.8%)	0.665^#^
no	65(65.0%)	76(76.0%)			38(73.1%)	36(69.2%)	

LM, left main coronary artery. LAD, left anterior descending branch. LCX, left circumflex branch. #, Chi-square test

**Table 3:** Univariate and multivariate Logistic regression analysis of RI in the near segment of LCX and the incidence of LM plaques before PSM

	Proximal LCX						LM				
	Univariate			Multivariate3			Univariate			Multivariate4	
	P	OR and 95%CI		P	OR and 95%CI		P	OR and 95%CI		P	OR and 95%CI
RI	0.090	1.705(0.921–3.157)		0.523	1.269(0.611–2.637)		0.164	1.545(0.837–2.854)		0.859	1.070(0.508–2.255)
∠LAD-LCX	0.220	1.007(0.996–1.018)		0.657	1.003(0.990–1.016)		0.250	1.006(0.996–1.017)		0.494	1.005(0.991–1.018)
Age	0.018	1.036(1.006–1.066)		0.026	1.034(1.004–1.066)		< 0.001	1.070(1.036–1.105)		< 0.001	1.070(1.035–1.106)
Diabetes	0.057	1.859(0.981–3.521)		0.100	1.751(0.898–3.412)		0.364	1.348(0.707–2.569)		0.437	1.319(0.656–2.653)

LCX, left circumflex branch. LM, left main coronary artery. Multivariate3 and Multivariate4: Corrects confounding factors age, diabetes, and ∠LAD-LCX.

**Table 4:** Univariate and multivariate Logistic regression analysis of RI in the incidence of plaques in the proximal segment of LAD before PSM

	Univariate			Multivariate1			Multivariate2	
	P	OR and 95%CI		P	OR and 95%CI		P	OR and 95%CI
RI	< 0.001	2.969(1.614–5.460)		0.023	2.147(1.113–4.143)		0.525	1.289(0.589–2.821)
∠LAD-LCX	< 0.001	1.022(1.010–1.035)		-	-		0.019	1.019(1.003–1.035)
Age	< 0.001	1.065(1.034–1.096)		< 0.001	1.065(1.032–1.099)		< 0.001	1.070(1.036–1.105)
Diabetes	0.001	3.463(1.663–7.212)		0.002	3.405(1.543–7.513)		0.003	3.367(1.504–7.537)

Multivariate1: Corrects confounding factors age and diabetes. Multivariate2: Corrects confounding factors age, diabetes, and ∠LAD-LCX.

### Comparison of general data and comparison of the incidence of plaques in bifurcation zone of the LCA among the RI distribution groups

The differences in general information (age, sex, history of hypertension, history of diabetes mellitus, history of hyperlipidemia, history of smoking, and ∠LAD-LCX angle) were not statistically significant when comparing the three groups (P > 0.05, see Table 5). the differences in the incidence of LAD proximal segment, LCX proximal segment, and LM plaque were not statistically significant in the lean-LAD group, lean-LCX group, and center group (P > 0.05, see Table 6).

**Table 5:** Comparison of general data among RI distribution groups

	Near LAD group (N = 16)	Near LCX group (N = 45)	Middle group (N = 39)	*P*
Age (years)	64.38 ± 9.65	60.76 ± 11.50	62.21 ± 9.45	0.482^*^
Gender (n%)				
Male	9(56.3%)	26(57.8%)	22(56.4%)	0.990^#^
Female	7(43.8%)	19(42.2%)	17(43.6%)	
Hypertension (n%)				
Yes	9(56.3%)	24(53.3%)	26(66.7%)	0.450^#^
No	7(43.8%)	21(46.7%)	13(33.3%)	
Diabetes (n%)				
Yes	6(37.5%)	16(35.6%)	19(48.7%)	0.451^#^
No	10(62.5%)	29(64.4%)	20(51.3%)	
Hyperlipidemia (n%)				
Yes	3(18.8%)	7(15.6%)	7(17.9%)	0.939^#^
No	13(81.3%)	38(84.4%)	32(82.1%)	
Smoking (n%)				
Yes	2(12.5%)	4(8.9%)	11(28.2%)	0.057^#^
No	14(87.5%)	41(91.1%)	28(71.8%)	
∠LAD-LCX(°)	92.50(26)	89.00(39)	92.00(40)	0.965^&^

LAD, left anterior descending branch. LCX, left circumflex branch. *, One-way ANOVA. #, Chi-square test. &, Nonparametric test

**Table 6:** Comparison of RI distribution and plaque incidence in bifurcation area of left coronary artery

	Near LAD group (N = 16)	Near LCX group (N = 45)	Middle group (N = 39)	*P*
LM(n%)				
Yes	5(31.3%)	13(28.9%)	16(41.0%)	0.488^#^
No	11(68.8%)	32(71.1%)	23(59.0%)	
Proximal LAD (n%)				
Yes	13(81.3%)	31(68.9%)	33(84.6%)	0.209^#^
No	3(18.8%)	14(31.1%)	6(15.4%)	
Proximal LCX (n%)				
Yes	4(25.0%)	12(26.7%)	19(48.7%)	0.071^#^
No	12(75.0%)	33(73.3%)	20(51.3%)	

LM, left main coronary artery. LAD, left anterior descending branch. LCX, left circumflex branch. #, Chi-square test

## Discussion

Current studies have shown that coronary artery anatomy is one of the risk factors affecting coronary atherosclerosis. Domestic and foreign scholars have concluded that the larger the left coronary artery bifurcation angle, the more likely plaque formation in the proximal segment of the left coronary artery bifurcation vessels [6, 9, 10].The generally accepted reason is that the increase in bifurcation angle causes a decrease in wall shear stress (WSS) and a change in laminar flow state, resulting in structural disturbances in the vascular endothelium, thus promoting plaque formation [11–13].The present study also confirmed that bifurcation angle is an independent risk factor for atherosclerosis in the proximal segment of LAD. The bifurcation angle of the RI group was also found to be significantly greater than that of the no-RI group, which is consistent with the Medrano-Gracia [14]. Whether the general increase in bifurcation angle in the RI population affects the occurrence of LAD plaques needs to be further investigated.

The hemodynamic effects of RI on the left main bifurcation zone and its correlation with the development of coronary atherosclerosis in the left main branch vessels are still controversial. Abuchaim [15]suggested that intermediate branches are present, usually without adverse hemodynamic effects, and may be protective against the development of myocardial ischemia. In contrast, Galbraith [16] showed that the presence of RI was associated with more proximal LAD lesions and larger anterior wall infarcts. They suggested that the presence of RI may have caused altered hemodynamics in the bifurcation zone, leading to increased eddy laminar flow in the proximal segment of the LAD (major branch vessels of the LM), which in turn promotes the development of atherosclerosis. In the present study, RI was found to be associated with proximal LAD plaque formation by both between-group difference comparison and one-way logistic regression analysis, and RI was a risk factor for proximal LAD plaque formation, in agreement with Galbraith and Rosani [17], but after controlling for confounding factors, the present study found that RI was not an independent risk factor for proximal LAD plaque, in addition Both intra-group comparisons of RI and between-group analysis after PSM revealed no statistical difference in the incidence of LAD plaque, further suggesting that the formation of proximal LAD plaque in the RI group is the result of a combination of factors interacting with each other. Risk factors influencing atherosclerosis in the left main trunk bifurcation zone are clinical (age, hypertension, diabetes mellitus, etc.) and anatomical (bifurcation angle, etc.), and the fact that bifurcation angle, an important independent risk factor, was not collected in the baseline data included in the study of Galbraith and Rosani may be the reason for the inconsistency with our results.

The incidence of LCX proximal segment plaques in this study was not statistically significant (P > 0.05) when compared between and within groups, which is consistent with the findings of El Zayat, Rosani, El Zayat [18]suggested that it was due to low longitudinal strain in LCX. In contrast to the results of the Rosani study, there was no statistical difference between the LM plaque groups in this study, which we believe may be equally influenced by the inconsistent risk factors included in both studies and needs to be further investigated in depth.

WSS is the friction force exerted on the surface of blood vessels as it moves through them. It is positively correlated with the formation of left coronary atherosclerotic plaque, and is also directly proportional to the flow velocity, viscosity, and flow rate, and inversely proportional to the cubic of the radius. At the same time, it is affected by a variety of factors such as the size of the bifurcation Angle [19]. The presence of RI may alter the anatomy of the left coronary artery bifurcation zone and the hemodynamics of this zone, which may be associated with atherosclerosis in the bifurcation zone, but further studies are needed to prove this. the LAD, as the main branch vessel of the left coronary artery, is undoubtedly the most affected [20].

RI is not an independent risk factor for atherosclerosis in the left coronary artery bifurcation zone. The formation of plaque in the bifurcation area of the LCA likely results from the actions of comprehensive factors, and RI may indirectly increase the risk of atherosclerosis in the proximal segment of the LAD.

This study had the following limitations: (1) This study was a single-centered retrospective observational study with a low level of evidences. (2) The sample size of this study was small, so the test efficiency was low. It is necessary to expand the sample size in future studies. (3) This study only included the confounding factors of hypertension history, diabetes history, hyperlipidemia history, and smoking history. Other confounding factors that affect the occurrence of plaques were not included, which may have biased the results of this study. (4) This study did not classify the nature of the plaques and the degree of vascular stenosis; these will be focused on in future studies.

## Data Availability

The datasets used and/or analysed during the current study available from the corresponding author on reasonable request.

## List of Abbreviation

CCTA: coronary computed tomography angiography
PACS: picture archiving and communication system
RI: ramus intermedius
PSM: propensity score matching
LCA: left coronary artery
LM: left main coronary artery
LAD: left anterior descending branch
LCX: left circumflex branch
MIP: maximum intensity projection
VR: volume rendering
SD: standard deviation
IQR: interquartile range
OR: odds ratio
WSS: wall shear stress
